# FIB-4 index is a marker for a subsequent decrease in insulin secretion in a non-diabetic Japanese population

**DOI:** 10.1038/s41598-020-72894-8

**Published:** 2020-09-25

**Authors:** Tomoyuki Fujita, Makoto Daimon, Satoru Mizushiri, Yuki Nishiya, Hiroshi Murakami, Jutaro Tanabe, Yuki Matsuhashi, Miyuki Yanagimachi, Itoyo Tokuda, Kaori Sawada, Kazushige Ihara

**Affiliations:** 1 Department of Endocrinology and Metabolism, Hirosaki University Graduate School of Medicine, 5-Zaifu-Cho, Hirosaki, Aomori 036-8562 Japan; 2 Department of Oral Healthcare Science, Hirosaki University Graduate School of Medicine, Hirosaki, Aomori Japan; 3 Department of Social Medicine, Hirosaki University Graduate School of Medicine, Hirosaki, Aomori Japan

**Keywords:** Disease prevention, Endocrine system and metabolic diseases, Predictive markers, Non-alcoholic fatty liver disease

## Abstract

Non-alcoholic fatty liver disease (NAFLD) is associated with a high risk of type 2 diabetes (DM), therefore, early diagnosis of NAFLD is important to prevent incident DM. FIB-4 index, a biomarker, often used to evaluate severity of NAFLD, may be useful to evaluate risk for incident DM in ordinary clinical setting. Here, we determined the association of FIB-4 index with changes in indices representing glucose metabolism with aging in a non-diabetic population. From among the participants of the population-based Iwaki study of Japanese people conducted during 2014–2017, 1,268 non-diabetic individuals with complete data sets (age: 51.4 ± 15.9 years; men/women: 485/773) were enrolled in a cross-sectional study. In addition, of the participants, 439 who attended consecutive appointments between 2014 and 2017 were enrolled in a longitudinal study that aimed to evaluate the changes in insulin secretion and resistance with aging (age: 53.1 ± 13.7 years; men/women: 178/261). The cross-sectional study showed significant correlations of FIB-4 index with homeostasis model of assessment (HOMA) indices, even after adjustment for multiple factors (HOMA-β: β =  − 0.254, p < 0.001; HOMA-R: β =  − 0.247, p < 0.001). The longitudinal study showed a significant association between FIB-4 index and the change in HOMA-β (p < 0.001) but not HOMA-R (p = 0.639) during the 3-year study period. Use of the optimal cut-off value of the FIB-4 index for the prediction of decreased insulin secretion (HOMA-β < 30), determined using receiver operating characteristic analysis (1.592), showed that individuals at risk had a hazard ratio of 2.22 (confidence interval 1.17−4.06) for decreased insulin secretion, after adjustment for confounders. FIB-4 index may represent a useful predictor of a subsequent decrease in insulin secretion, at least in a non-diabetic Japanese population.

## Introduction

Type 2 diabetes (DM) increases the risks of serious physical and mental health problems, and, the prevalence of DM is increasing worldwide^1,2^. Therefore, finding individuals at risk to develop abnormality of glucose metabolism or DM seems to be an important issue to prevent an increase of DM. For such purpose, biological markers seem to be useful in ordinary clinical settings, whether their associations are cause or consequence, or merely association. To date, since no efficient marker besides those directly related to glucose metabolism such as glucose levels per se and glycated substrates, is in clinical practice, finding such markers need to be awaited.

Since DM is a heterogeneous disorder of glucose metabolism that is characterized by both insulin resistance and pancreatic β-cell dysfunction^3,4^, in addition to the pancreas, the liver also plays a pivotal role in its pathophysiology^3,4^. Liver dysfunction in diabetic individuals is thought to be mainly caused by non-alcoholic fatty liver disease (NAFLD), and close associations between NAFLD and DM have been reported many times^5–7^. In addition, meta-analyses have shown that there is about a two-fold higher risk of DM developing in individuals with NAFLD^8,9^. Therefore, NAFLD can be regarded as a cause of DM, and therefore early diagnosis of NAFLD may be important to prevent the development of DM or the progression of abnormal glucose metabolism. Moreover, although the influence of the progression of NAFLD or simple steatosis to non-alcoholic steatohepatitis (NASH) on the association with DM or glucose metabolism has not been thoroughly studied, previous studies have shown close associations between decreased glucose tolerance and the presence and severity of liver fibrosis, rather than the degree of steatosis^7,10^. Therefore, evaluation of not only the presence, but also the severity, of NAFLD appears to be important. The diagnosis of NAFLD is made on the basis of the available methodology, and in ordinary clinical setting, liver biopsy, the gold standard method of evaluating the severity of NAFLD, cannot be easily performed. Therefore, optimal methods of not only determining the presence, but also evaluating the severity of NAFLD, still remain to be identified.

Several biomarkers have been proposed for the evaluation of fibrosis in the liver. Among these, the fibrosis (FIB)-4 index has been most thoroughly validated, as shown by studies of patients with ultrasonography-diagnosed NAFLD^11,12^. Therefore, FIB-4 index may be a useful marker of evaluating the severity of NAFLD, and thus may also be useful for evaluation of the risk for incident DM or the progression of abnormal glucose metabolism in the general population. Furthermore, evaluation of the relationship between FIB-4 index and changes in glucose metabolism in the general subjects may provide evidence of the mechanisms whereby NAFLD predisposes toward DM, which is generally though to be via an increase in insulin resistance^5–7^. Therefore, in the present study, we evaluated the relationships between FIB-4 index and changes in indices of glucose metabolism, or insulin secretion and resistance over a 3-year period, as well as evaluating the cross-sectional relationship between FIB-4 index and insulin secretion and resistance, in a non-diabetic Japanese population, which, like other Asian populations, demonstrates lower insulin secretion and resistance than other ethnicities^13^.

## Material and methods

### Study populations

In the present study, we used two different samples for a cross-sectional and a longitudinal study. The participants in both were recruited from the Iwaki study, a health promotion study of Japanese people over 20 years of age that aimed to prevent lifestyle-related diseases and to prolong lifespan. The Iwaki study is conducted annually in the Iwaki area of Hirosaki city in Aomori Prefecture in northern Japan^14,15^. Of the 1,597 participants in the Iwaki study conducted in 2014–2017, 1,268 non-diabetic individuals with complete data sets and fasting blood glucose (FBG) concentrations > 63 mg/dl (to precisely evaluate homeostasis model of assessment (HOMA) indices) (age: 51.4 ± 15.9 years; men/women: 485/773), were enrolled in the cross-sectional study. Furthermore, to evaluate the changes in HOMA indices, representing insulin secretion and resistance, with aging, of the 555 participants who attended consecutive appointments between 2014 and 2017, 439 non-diabetic individuals with complete data sets and FBG concentrations > 63 mg/dl (age: 53.1 ± 13.7 years; men/women: 178/261) at baseline, or in 2014, were enrolled in the longitudinal study. In addition, individuals on treatment for hyperlipidemia were excluded throughout to better avoid possibility of the treatment effects on glucose metabolism or HOMA indices.

This study was approved by the Ethics Committee of the Hirosaki University School of Medicine (No. 2014-014, 2014-377, 2016-028, and 2017-026), and was conducted according to the guidelines of the Declaration of Helsinki. Written informed consent was obtained from all the participants.

### Parameters measured

Blood samples were collected in the morning from a peripheral vein of fasted participants. The following parameters were measured: height; body weight; body mass index (BMI); percentage body fat (fat (%)); fasting blood glucose (FBG); fasting serum insulin; glycated hemoglobin (HbA1c); systolic and diastolic blood pressure; serum low-density lipoprotein (LDL)-cholesterol, triglyceride (TG), high-density lipoprotein (HDL)-cholesterol, uric acid, urea nitrogen, creatinine, and albumin concentrations; aspartate transaminase (AST), alanine transaminase (ALT), and γ-glutamyl transpeptidase (γGTP) activities; and blood platelet count. Fat (%) was measured using the bioelectricity impedance method with a Tanita MC-190 body composition analyzer (Tanita Corp., Tokyo, Japan). HbA1c (%) is expressed as the National Glycohemoglobin Standardization Program value. All laboratory tests were performed in a commercial laboratory (LSI Medience Co., Tokyo, Japan) according to the manufacturer’s protocols. Insulin secretion was evaluated using HOMA of β-cell function (HOMA-β), which is calculated using fasting blood glucose and serum insulin concentrations^16^. Insulin resistance was evaluated using HOMA-R^15^. FIB-4 index was calculated as (age × AST)/(blood platelet count × √ALT)^17^. Onset and/or treatment for diseases including diabetes were asked with a questionnaire. DM was defined using to the criteria of the Japan Diabetes Society published in 2010: FBG ≥ 126 mg/dl^18^. In participants in whom FBG concentration was not measured, diabetes was defined as HbA1c ≥ 6.5%. Hypertension was defined as a blood pressure of ≥ 140/90 mmHg or current treatment for hypertension. Hyperlipidemia was defined as an LDL-cholesterol of ≥ 140 mg/dl, a TG of ≥ 150 mg/dl. Alcohol consumption (current or non-current) and smoking habits (never, past or current) were determined using questionnaires.

### Statistical analysis

Data are presented as means ± SDs. The statistical significance of the differences in values between two groups (parametric) and case–control associations between groups (nonparametric) were assessed using the Student's t-test and the χ^2^ test, respectively. The relationships between clinical parameters and HOMA indices were evaluated using univariate or multivariate (for independent associations) linear regression analyses. For multivariate analyses, among characteristics related together, one characteristic which showed highest correlation coefficient was selected as an independent variable: e.g. one out of systolic and diastolic blood pressure. To evaluate the effects of aging on HOMA indices and to identify the factors affecting the changes in these indices with aging, repeated–measures analysis of covariance (repeated measures-ANOVA) was used. Decreased insulin secretion was tentatively defined here as HOMA-β < 30, since the definition is popularly used for Japanese subjects^19^, who demonstrate lower insulin secretion and resistance than other ethnicities^20^. The risk of a development of decreased insulin secretion, defined as HOMA-β < 30, was evaluated using Kaplan–Meier and multivariate Cox proportional hazard regression analyses. Cox proportional hazard regression models were used to calculate hazard ratios (HR) for the FIB-4 index at baseline for a development of decreased insulin secretion, after adjustment for factors found to be affecting the change in HOMA-β with aging (e.g. fat (%)). Receiver operator characteristic (ROC) curves were plotted to determine the FIB-4 index cutoff value at baseline that was most appropriate for the identification of individuals who were at risk of a development of decreased insulin secretion. The value that yields the greatest sensitivity and specificity was determined as the cut-off value. Prior to statistical analysis, HOMA indices, TG, γGTP, and FIB-4 index were log-transformed (log10) to approximate a normal distribution. p < 0.05 was considered to represent statistical significance. All analyses were performed using JMP version 14.0 (SAS Institute Japan Ltd., Tokyo, Japan).

## Results

### Clinical characteristics of the participants

The clinical characteristics of participants in the cross-sectional study and the baseline characteristics of those included in the longitudinal study are shown in Table 1. The mean ages of the participants were 51.4 ± 15.9 and 53.1 ± 13.7, respectively (p = 0.577). No clinical characteristics, including HOMA indices, differed between the two groups.

**Table 1:** Clinical characteristics of the study subject.

Characteristics	Cross-sectional	Longitudinal	p
Number (gender: M/F)	1268 (485/773)	439 (178/261)	0.577
Age (years)	51.4 ± 15.9	53.1 ± 13.7	0.051
Body mass index (kg/m^2^)	22.6 ± 3.4	22.5 ± 3.2	0.817
Fat (%)	24.7 ± 8.2	23.9 ± 7.7	0.073
Systolic blood pressure (mmHg)	127.0 ± 19.7	127.5 ± 18.9	0.639
Diastolic blood pressure (mmHg)	76.7 ± 11.9	77.6 ± 11.2	0.145
FIB-4 index	1.21 ± 0.70	1.22 ± 0.59	0.095
AST (IU/L)	22.4 ± 29.8	21.5 ± 7.1	0.538
ALT (IU/L)	20.5 ± 21.9	20.4 ± 13.3	0.892
γGTP	30.5 ± 38.8	28.4 ± 20.3	0.815
Blood pletelet count (10^9^/L)	23.5 ± 5.6	23.2 ± 5.2	0.252
HbA1c (%)	5.62 ± 0.30	5.65 ± 0.34	0.059
Fasting plasma glucose (mg/dl)	80.6 ± 10.2	80.1 ± 9.1	0.328
Fasting serum insulin:IRI (mU/ml)	4.5 ± 2.6	4.2 ± 2.1	0.111
HOMA-R	0.90 ± 0.60	0.85 ± 0.46	0.265
HOMA-β	134.3 ± 167.8	123.7 ± 142.3	0.562
Serum albumin (g/dl)	4.5 ± 0.3	4.5 ± 0.3	0.432
LDL cholesterol (mg/dl)	115.9 ± 29.1	117.6 ± 28.1	0.283
Triglyceride (mg/dl)	94.2 ± 71.7	93.8 ± 64.2	0.902
HDL cholesterol (mg/dl)	65.2 ± 16.7	65.9 ± 17.1	0.437
Serum uric acid (mg/dl)	4.9 ± 1.4	4.9 ± 1.4	0.794
Serum urea nitrogen (mg/dl)	14.4 ± 4.3	14.7 ± 4.3	0.229
Serum creatinine (mg/dl)	0.69 ± 0.15	0.70 ± 0.16	0.914
Hypertension: n (%)	472 (37.2)	168 (38.3)	0.697
Hyperlipidemia: n (%)	356 (28.1)	130 (29.6)	0.539
Drinking alcohol: n (%)	568 (44.9)	223 (46.2)	0.618
Smoking (never/past/current): n	767/255/244	274/99/66	0.116

Data are mean ± SD or number of subjects (%).

### Correlations with insulin secretion and resistance (HOMA indices)

The univariate correlations between the clinical characteristics and HOMA indices (β and R) are shown in Table 2. In addition to many clinical characteristics, such as age, gender, BMI, fat (%), blood pressures, HbA1c, and serum lipid, uric acid, and urea nitrogen concentrations, FIB-4 index correlated with HOMA indices, and the strongest correlation among these was for HOMA-β (β = − 0.445) (Supplemental Fig. 1). The correlations between FIB-4 index and HOMA indices were remained significant after adjustment for the characteristics found to be correlated in univariate analyses (HOMA-β: β = − 0.254, p < 0.001; HOMA-R: β = − 0.247, p < 0.001).

**Table 2:** Factors correlated with HOMA indices in the subjects of the cross sectional study.

	HOMA-β				HOMA-R			
	Univariate		Multivariate^#^		Univariate		Multivariate^#^	
Characteristics	β	p	β	p	β	p	β	p
Gender (F)	0.124	< 0.001	0.064	0.097	0.052	0.066	0.186	< 0.001
Age (years)	− 0.405	< 0.001	− 0.151	0.003	0.030	0.288	0.085	0.073
Body mass index (kg/m^2^)	0.167	< 0.001	–	–	0.496	< 0.001	0.389	< 0.001
Fat (%)	0.225	< 0.001	0.200	< 0.001	0.455	< 0.001	–	–
Systolic blood pressure (mmHg)	− 0.163	< 0.001	0.010	0.707	0.150	< 0.001	–	–
Diastolic blood pressure (mmHg)	− 0.051	0.071	–	–	0.161	< 0.001	0.040	0.115
FIB-4 index	− 0.445	< 0.001	− 0.254	< 0.001	− 0.131	< 0.001	− 0.247	< 0.001
AST (IU/L)	− 0.090	0.001	–	–	− 0.078	0.006	–	–
ALT (IU/L)	− 0.002	0.953	–	–	0.070	0.012	–	–
γGTP	− 0.056	0.046	0.067	0.024	0.102	< 0.001	0.023	0.463
Blood pletelet count (10^9^/L)	0.200	< 0.001	–	–	0.072	0.011	–	–
HbA1c (%)	− 0.286	< 0.001	− 0.184	< 0.001	0.217	< 0.001	0.129	< 0.001
Serum albumin (g/dl)	0.093	< 0.001	–	–	0.081	0.004	–	–
LDL cholesterol (mg/dl)	− 0.067	0.016	− 0.009	0.723	0.199	< 0.001	0.005	0.847
Triglyceride (mg/dl)	0.113	< 0.001	–	–	0.305	< 0.001	0.184	< 0.001
HDL cholesterol (mg/dl)	− 0.145	< 0.001	− 0.122	< 0.001	− 0.267	< 0.001	–	–
Serum uric acid (mg/dl)	− 0.006	0.83	–	–	0.138	< 0.001	0.062	0.043
Serum urea nitrogen (mg/dl)	− 0.266	< 0.001	− 0.022	0.403	0.021	0.463	–	–
Serum creatinine (mg/dl)	− 0.046	0.098	–	–	0.039	0.167	–	–
Drinking alcohol: n (%)	− 0.126	< 0.001	− 0.102	< 0.001	− 0.149	< 0.001	− 0.111	< 0.001
Smoking (never/past/current): n	0.027	0.330	–	–	− 0.071	0.0120	− 0.057	0.030

**Figure 1:**
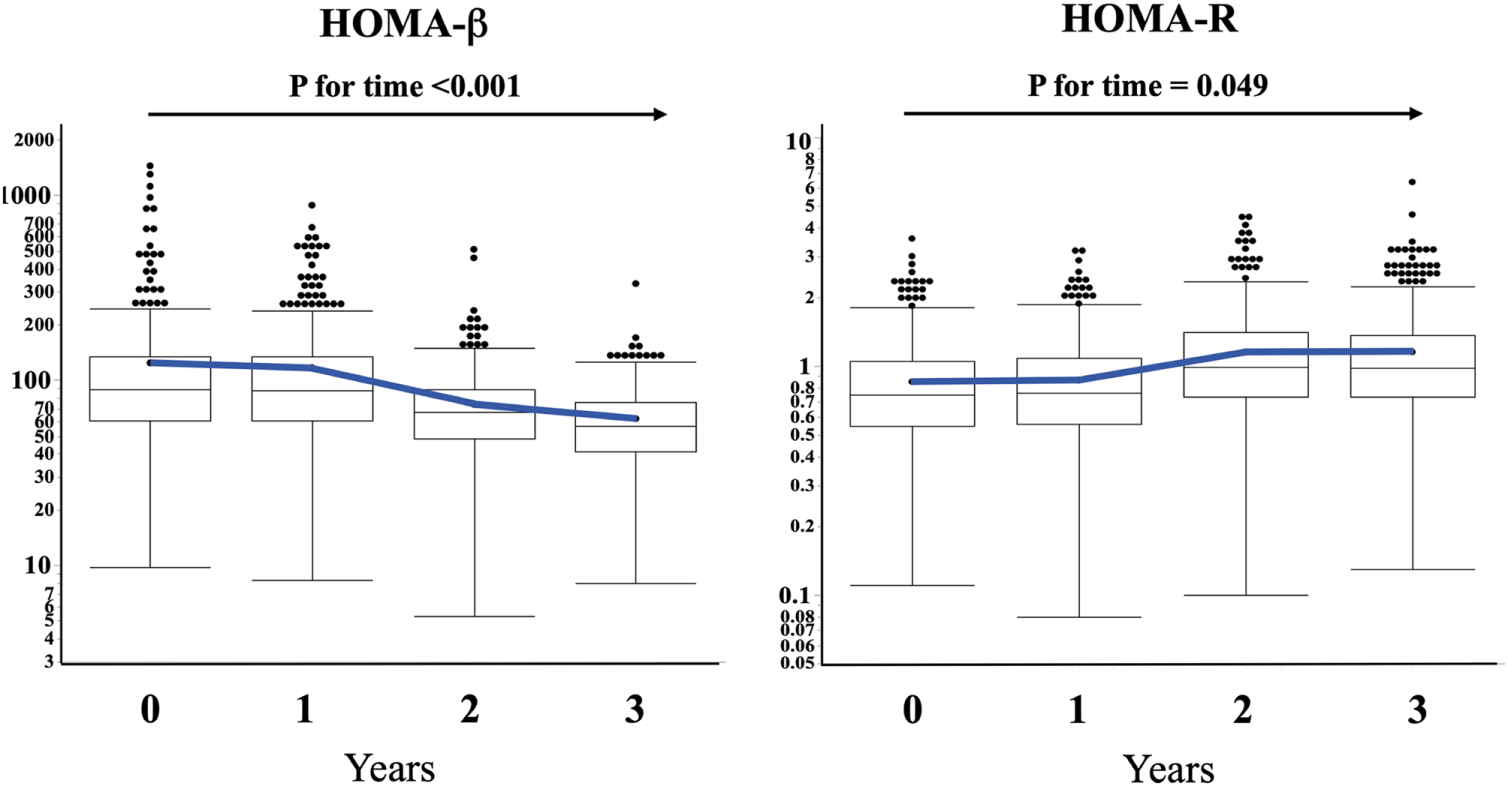
Changes in HOMA indices over time or with aging. Individual data at each time point are shown in a box-and-whisker plot, with the mean values connected with a line. P-values for time evaluated using repeated–measures analysis of covariance with adjustment for the characteristic included in the multivariate analyses of the cross-sectional sample are indicated on each panel.

### Relationship between FIB-4 index and changes in insulin secretion and resistance (HOMA indices) with aging

As shown, although correlations between the HOMA indices and age in the cross-sectional study were different depending on HOMA indices, HOMA-β with age even after adjustment for the characteristics found to be correlated in univariate analyses (HOMA-β: β =  − 0.151, p = 0.003; HOMA-R: β = 0.085, p = 0.073) (Table 2). We then evaluated the relationship between FIB-4 index and changes in HOMA indices with aging in the longitudinal study participants. Repeated-measures ANOVA showed that HOMA-β decreased significantly with aging (over time) (p < 0.001), while HOMA-R increased significantly (p = 0.049) (Fig. 1). Furthermore, of the characteristic adjusted for in the analysis of the cross-sectional sample (Table 2), FIB-4 index was found to associate significantly with change in HOMA-β(time) (p < 0.001) not with change in HOMA-R (time) (p = 0.639) during the 3-year period ) (Table 3).

**Table 3:** Factors related with changes in HOMA indices with aging in the subjects of the longitudinal study.

HOMA-β		HOMA-R	
Characteristics	p	Characteristics	p
FIB-4 index	< 0.001	FIB-4 index	0.639
Gender (F)	0.242	Gender (F)	0.309
Age (years)	0.948	Age (years)	0.227
Fat (%)	0.078	Body mass index (kg/m^2^)	0.540
HbA1c (%)	0.311	HbA1c (%)	0.050
γGTP	0.650	Triglyceride (mg/dl)	0.820
HDL cholesterol (mg/dl)	0.608	Serum uric acid (mg/dl)	0.339
Drinking alcohol: n (%)	0.067	Drinking alcohol: n (%)	0.177
–	–	Smoking (never/past/current): n	0.363

p values for association between the factors and the change in HOMA indices during the 3-year period are shown.

### High FIB-4 index is associated with a higher risk of a development of decreased insulin secretion

**Figure 2:**
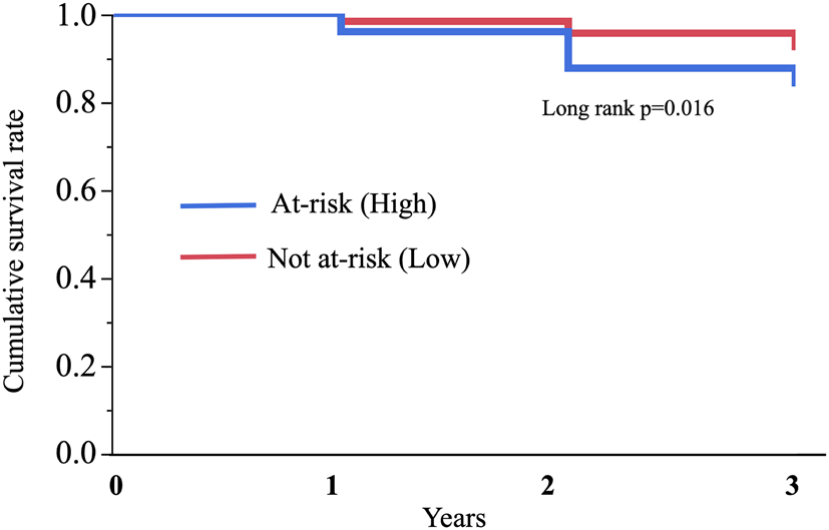
Kaplan–Meier survival curves for the participants, stratified into two groups on the basis of FIB-4 index at baseline. Use of the optimal cut-off value of the FIB-4 index for the prediction of decreased insulin secretion (HOMA-β < 30) determined using receiver operating characteristic analysis (1.592), the subjects in the longitudinal study were stratified into two groups: an at-risk (high) and a not at-risk (low) groups. The differences between the groups were assessed using log-rank test. *p* < 0.05 was considered to represent statistical significance.

To evaluate the relationship between FIB-4 index and a decrease in HOMA-β or insulin secretion with aging more, we then examined the risk that FIB-4 index presents for a development of decreased insulin secretion, defined as HOMA-β < 30, using the data from the participants in the longitudinal study, but excluding those with decreased insulin secretion at the baseline (n = 10). For this analysis, a ROC curve was plotted to determine the optimal cut-off value for FIB-4-index at baseline for the prediction of a development of decreased insulin secretion using the data from the participants in the cross-sectional study. In the ROC curve analysis (area under the curve: 0.726), FIB-4-index of 1.592, yielded the greatest sensitivity and specificity (78.3 and 62.0%, respectively). Then, the subjects in the longitudinal study were stratified into two groups using this cut-off value: an at-risk (high) and a not at-risk (low). During the 3-year period of the study, in 44 (10.3%) of the participants, HOMA-β decreased to < 30 (decreased insulin secretion). The numbers of participants reached the endpoint (decreased insulin secretion) during the 3-year period in the at-risk and the not at-risk groups were 16/96 (16.7%) and 28/333 (8.4%), respectively (odds ratio of the at-risk group was 2.18 (95% confidence interval (CI) 1.12–4.22)). Analysis using the Kaplan–Meier method showed a significantly higher risk of a development of decreased insulin secretion in the at-risk group (log rank p = 0.016) (Fig. 2). Cox’s proportional hazard regression model analysis also showed impacts of an increase in FIB-4 index and the at-risk group on their risk of a development of decreased insulin secretion, which remained significant after adjustment for gender (hazard ratio (HR) (95% confidence interval): 19.61 (4.50–87.05) and 2.22 (1.17–4.06), respectively) (Table 4). Further adjustment with characteristics shown to be correlate with HOMA-β, i.e. gender, fat (%), HbA1c, γGTP, HDL-cholesterol, and drinking alcohol, also showed an increase in FIB-4 index as a risk of a development of decreased insulin secretion (HR: 16.85, CI 3.20–93.29). In addition, although such adjustment made the risk of the at-risk group insignificant (HR: 1.89, CI 0.97–3.54), addition of individuals on treatment for hyperlipidemia at the baseline to increase the sample size (n = 479) kept the risk significant (HR: 1.98, CI 1.06–3.61).

**Table 4:** Risk of FIB-4 index for a development of decreased insulin secretion (HOMA-β < 30) in the subjects of the longitudinal study.

	Univariate			Adjusted with gender		
	HR	95%CI	p	HR	95%CI	p
FIB-4 index (per 1 logx10)	17.40	3.81–81.94	< 0.001	19.61	4.50–87.05	< 0.001
High (FIB-4 index ≥ 1.592) versus low	2.07	1.09–3.78	0.026	2.22	1.17–4.06	0.015

Risks of an increase in FIB-4 index and the at-risk group were separately evaluated using Cox proportional hazard regression model analysis.

HR, hazard ratio, CI, confidence interval.

Taken together, these findings indicate that a higher FIB-4 index is a risk factor for a subsequent decrease in insulin secretion, independent of a change in insulin secretion with aging.

## Discussion

In the study of a non-diabetic, healthy Japanese population, we found that higher FIB-4 index is a risk for a subsequent decrease in insulin secretion. This implies that FIB-4 index may represent a predictor for the risk of developing DM in non-diabetic Japanese people. This finding is not consistent with those of previously published studies, in which NAFLD was shown to increase insulin resistance, with a compensatory increase in insulin secretion^21,22^, which is eventually followed by the development of glucose intolerance or DM when this compensatory mechanism fails^5–7^, while relationships of FIB-4 index with HOMA-β but not with HOMA-R were recently reported in a population-based cross-sectional study of Japanese^23^. Although we have no precise explanation for this inconsistency, differences in study samples may be responsible. The participants in the present study were non-diabetic, recruited from a health promotion study, and, thus, appear to be very healthy, as their BMI, HOMA-β, and HOMA-R were 22.6, 134.3, and 0.90, respectively (Table 1). This may explain why HOMA-R changed only modestly during the 3-year period of the study (Fig. 1 and Table 3) and, therefore, the relationship between FIB-4 index and the change in HOMA-R might not be properly evaluated. Nevertheless, because HOMA-β decreased significantly during the study period, the identified association between FIB-4 index and HOMA-β is likely to be more reliable. Furthermore, as previously described, Asian people show lower insulin secretion and resistance than other ethnicities^13^, and therefore the decreases in insulin secretion might be involved more substantially than an increase in insulin resistance in the pathogenesis of abnormal glucose metabolism, at least in the non-diabetic, non-obese, healthy Japanese population.

Although associations between NAFLD and incident DM have been frequently reported^6,7^, associations between the FIB-4 index and incident DM or abnormal glucose metabolism have rarely been reported. A longitudinal study of patients with biopsy-proven NAFLD showed that FIB-4 index did not predict the development of DM^24^. Furthermore, a longitudinal study of non-obese individuals also showed that FIB-4 index alone did not predict incident DM after adjustment for multiple factors^25^. NAFLD comprises two principal components, steatosis and fibrosis, and the FIB-4 index describes the severity of hepatic fibrosis, but not steatosis. Fat accumulation in the liver is an important risk factor for insulin resistance, and therefore DM^21,26^, which may explain why FIB-4 index has not been shown to be associated with incident DM previously. Although steatosis can be estimated by several indices such as Fatty liver index and Zhejiang University index^27,28^, we did not evaluated such indices here, and, thus, the above mentioned issue needs to be examined with such indices in the future.

Here, we have shown an association between FIB-4 index and a development of decreased insulin secretion, rather than with incident DM per se, as the number of subjects in the longitudinal study (n = 439) appeared to be small for such analyses (incidence of diabetes during the 3-year period was 9). Nevertheless, although not significant (p = 0.088), the subjects who had incident diabetes during the 3-year period had higher FIB-4 index at the baseline (1.46 ± 0.31) than the others (1.22 ± 0.59). As described previously, because the main feature of the pathogenesis of DM that is linked to NAFLD is insulin resistance, the impact of a decrease in insulin secretion on incident DM may not be substantial, and therefore may be missed in the studies with small sample size and/or of short duration. Nevertheless, the lack of association between FIB-4 index and the change in insulin resistance identified during the 3-year study period is, at least in part, in accordance with previous findings. To our knowledge, no previous study has aimed to determine the relationship between FIB-4 index and the change in insulin secretion or a decreased insulin secretion, which makes the present findings novel, although further larger and longer studies should be conducted in other, especially non-Asian, populations.

Although we here reported that the at-risk group defined based on the cut-off value, 1.592, of the FIB-4 index is a risk for a subsequent decrease in insulin secretion, the value itself seems to be tentative, since the clinical relevance of the value was not evaluated in this study. However, during the 3-year period of the study, the numbers of participants reached the endpoint in the at-risk and the not at-risk groups were 16.7% and 8.4%, respectively, and therefore the value may have, at least, some clinical relevance. Anyway, such values should be evaluated in consideration with clinical relevance in the future with much larger sample of general Japanese population with longer follow-up period.

The mechanisms underpinning the association between high FIB-4 index and a decreased insulin secretion are uncertain if insulin resistance is not the key mediator. High FIB-4 index may indicate that the patient has had NAFLD for longer, and has therefore been exposed to metabolic or other insults that affect insulin secretion for a longer period of time. For example, increased free fatty acids and their related compounds such as ceramides and diacylglycerols often observed in NAFLD can impair insulin secretion (known as lipotoxicity)^7,29,30^. Dietary energy restriction causes a reduction in the fat content of both in the liver and the pancreases, which can normalize β-cell function and hepatic insulin sensitivity in diabetic subjects^31,32^. Therefore, extent of steatosis of the liver or the severity of NAFLD may, at least in part, mirror the effects of pancreatic steatosis.

The present study had several strengths and limitations. With regard to its strengths, we studied a sample from the general population and accounted for multiple factors that could have confounded the statistical analyses. Furthermore, we conducted a longitudinal study, as well as a cross-sectional study, and could therefore evaluate the relationship between FIB-4 index and a subsequent development of decreased insulin secretion. Moreover, we excluded diabetic participants and those with FBG concentrations ≦ 63 mg/dl to permit more precise evaluation of the HOMA indices. Therefore, the results obtained should accurately reflect the relationship between FIB-4 index and HOMA indices. However, these exclusions, together with the recruitment of participants from a health promotion study, rather than from a population attending health checks, may also represent a limitation. Indeed, female proportion is substantially high (61.0%). Namely, the participants may have been more invested in their health than the general population, and therefore may not accurately represent the general population. In addition, the presence of NAFLD was not evaluated using any methods other than FIB-4 index, which is not a marker of liver steatosis, but of liver fibrosis alone^17,33^. Therefore, we evaluated the severity of NAFLD, but not its presence per se. Furthermore, although FIB-4 index has been developed to evaluate the liver fibrosis in the chronic liver disease including viral hepatitis and NAFLD and well validated by comparing it with findings of liver biopsy, it still remains unclear whether FIB-4 index represents liver fibrosis in the subjects without definite chronic liver disease, and therefore using FIB-4 index in the study with such healthier subjects may not be adequate. However, FIB-4 index was significantly correlated with γGTP in this study population (β = 0.090, p = 0.036, after adjustment with age and gender), and, thus, can be, at least, applied for this study. Alternatively, the observed association between higher FIB-4 index and a development of decreased insulin secretion may not be via liver fibrosis per se. Moreover, presence and/or incidence of any other liver diseases were not considered, as any markers other than AST, ALT, and γ-GTP activities or markers of viral hepatitis and autoimmune liver diseases were not measured, and therefore studies with such considerations are warranted. Besides, as described, since we did not determine the presence of NAFLD per se by any methods for the diagnosis such as ultrasonography and/or transient elastography, the results obtained cannot be regarded in association with NAFLD per se. Therefore, without such precise diagnosis of NAFLD, no mechanisms involved in the observed association can be precisely evaluated. However, the study is to evaluate association between FIB-4 index, not NFLD per se, and a subsequent development of decreased insulin secretion or DM, and, thus, the results per se seem to be, at least, reliable. Finally, the 3-year follow-up period may be short for this kind of longitudinal study. However, since the number of individuals who attended consecutive appointments becomes smaller if we chose longer follow-up period, we did not choose longer follow-up period to have sufficient statistical power. Nevertheless, in the 3-year follow-up period, significant difference between the groups was found, and, thus, the follow-up period seemed to be sufficient, at least, for the analysis.

In conclusion, FIB-4 index may represent a useful predictor of a subsequent development of decreased insulin secretion or DM, at least in a non-diabetic, apparently healthy Japanese population.

## Supplementary information

Supplementary information.

## Data Availability

All data generated or analyzed during this study are included in this published article.
