# Supplementary material for: FIB-4 index is a marker for a subsequent decrease in insulin secretion in a non-diabetic Japanese population
Source: Sci Rep. 2020 Sep 25;10:15814. doi: 10.1038/s41598-020-72894-8 (PMC7519131; doi:10.1038/s41598-020-72894-8)

**Supplementary Figure 1.**

**Correlations between FIB-4 index and HOMA indices**

Linear regression lines are shown. Correlation coefficients and *p*-values are indicated on each panel. *P* < 0.05 was considered to represent statistical significance.


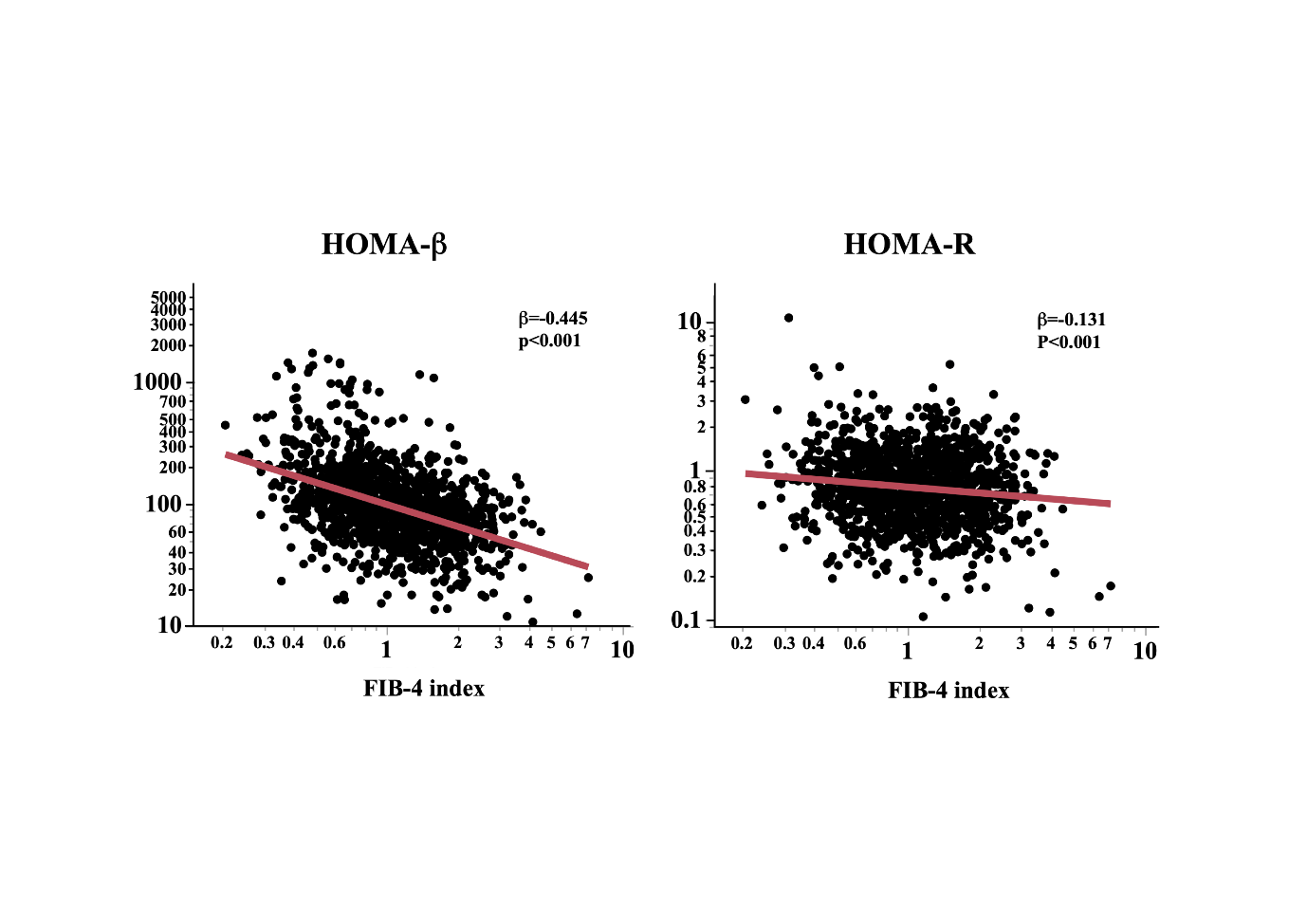

Supplement: Supplementary file 1 — Supplementary information. [file 41598_2020_72894_MOESM1_ESM.docx]
